# Perceptions of cannabis among adults aged 60 years and older in Canada: a qualitative study

**DOI:** 10.24095/hpcdp.45.10.01

**Published:** 2025-10

**Authors:** Justine Renard, Balpreet Panesar, Sima Noorbakhsh, Elle Wadsworth, Nick Cristiano, Robert Gabrys

**Affiliations:** 1 Canadian Centre on Substance Use and Addiction, Ottawa, Ontario, Canada; 2 Institute for Better Health, Trillium Health Partners, Mississauga, Ontario, Canada; 3 CHU Sainte-Justine Research Centre, University of Montral, Quebec, Canada; 4 RAND Europe, Eastbrook, Cambridge, United Kingdom; 5 Trent University, Oshawa, Ontario, Canada; 6 Department of Neuroscience, Carleton University, Ottawa, Ontario, Canada

**Keywords:** cannabis, older adults, legalization, public health, Canada

## Abstract

**Introduction:**

Since cannabis legalization in Canada, consumption by older adults has risen more rapidly than in other age groups. There is a need to better understand patterns of consumption, motivations, access, perceptions of risks and benefits, and how legalization has changed older adults’ behaviours, especially across gender, and frequency of use.

**Methods:**

We conducted 10 online focus groups with 72 participants aged 60 years and older, segmented by cannabis use frequency. Focus groups were held across five regions in Canada. Data were collected using open-ended questions and analyzed thematically.

**Results:**

Analysis revealed five themes: common practices; general knowledge; perceived harms; perceived benefits; and changes in stigma and social acceptability following legalization. The participants used various consumption methods, primarily oral consumption of edibles (gummies, capsules and baked goods) and inhalation (vaping and smoking). Legalization may have decreased stigma associated with cannabis use. Both frequent and infrequent consumers noted the therapeutic benefits of cannabis, particularly for pain management and mental health, but many expressed concerns about potential physical and cognitive adverse effects, possible interactions with medications and a lack of trustworthy sources of information or guidance from health care providers.

**Conclusion:**

The findings demonstrate the complexities of cannabis consumption among older adults, who have specific challenges and risks, and the need for comprehensive public education and support from health care providers. Targeted research and policy development to address the specific needs of this underrepresented population are urgently needed.

**Highlights**

- We investigated the experiences, behaviours and perceptions of cannabis consumption among adults aged 60 years and older in Canada.
- Older adults consume cannabis for many reasons, including for physical and mental health and recreationally.
- There are gender differences in cannabis consumption, with females preferring edibles and topicals, and males preferring smoking and vaping.
- Both frequent and infrequent consumers worried about the physical harms of cannabis consumption, in particular the potential cognitive decline and the effects of smoking on the lungs.
- Despite legalization, cannabis-related stigma persists for older adults, although perceptions of stigma differ between frequent and infrequent consumers.

## Introduction

Cannabis for medical purposes became formally available to people living in Canada in 2001. Authorization by a health care provider allowed individuals with specific medical conditions to obtain cannabis legally through licensed producers, to register to grow cannabis themselves or to designate another individual to produce it on their behalf.^1^ In October 2018, Canada implemented the *Cannabis Act*, which legalized nonmedical (or recreational) cannabis use nationwide for adults aged 18years and older.^2^ This made Canada the second country in the world, after Uruguay, to legalize the use of nonmedical cannabis.

Acceptance of cannabis has progressively increased across all age groups, continuing a trend that began before legalization and reflecting broader changes in how people perceive cannabis and its risks and benefits.^3^-^5^ Studies have shown that legalization has influenced older adults’ beliefs and perceptions about cannabis, contributed to its destigmatization, fostered more positive attitudes toward it and increased its acceptance.^4^-^8^

These shifts in perceptions and behaviours among older adults were also reflected in national surveys. For example, the 2019 National Cannabis Survey found that adults aged 65 years and older were the fastest-growing population of cannabis consumers, increasing from less than 1% in 2012 to 6.6% in 2019, with 27.0% reporting first-time use within the past 3months.^9^ Similarly, the International Cannabis Policy Study found that past 12-month cannabis use significantly increased among adults aged 55 to 65 years, from 19.3% in 2018 to 24.5% in 2019, the first year after legalization, and has remained stable since (24.3% in 2020 and 25.6% in 2021).^10^ This study also found that a large proportion of older individuals who use cannabis do so for physical or mental health reasons rather than recreationally.^10^

A public opinion study found that older Canadians perceived cannabis use to be relatively common, with many reporting using it for medical purposes, especially to manage pain, stress and sleep, as well as for recreational purposes.^11^ Aside from these findings, little is known about cannabis use by older individuals, highlighting the need for targeted studies to understand and address the unique needs of this underrepresented population.

Cannabis use can have significant risks, including for new older adult consumers. First, there may be a discrepancy between older adults’ perceptions of cannabis potency, based on past experiences, and the more potent products currently on the market. Dried cannabis products in Canada can contain up to 30% delta-9-tetrahydrocannabinol (THC), while chemically concentrated extracts (e.g. shatter, budder, wax) can contain up to 90% THC.^12^ A recent systematic review found that the use of high-potency cannabis products, defined by the authors as concentrates (THC ≥60%), resin or hash (THC about 30%–50%), and high-potency herbal cannabis (THC 20%–30%) was generally associated with poorer mental health, problematic cannabis use and polysubstance use, although most of the included studies were of low quality.^13^

The consumption of high-potency cannabis products can lead to increased blood pressure, heart rate and anxiety and to dizziness and falls among older adults.^14^-^17^ The variety of consumption methods, including smoking, vaping and oral consumption, may make managing dosages and understanding onset times more difficult, potentially resulting in unintentional overconsumption. 

Other concerns include multimorbidity, interactions with existing medications and altered metabolic processing. Older adults have greater risk of chronic conditions (e.g. chronic pain, insomnia, and mood and cognitive disorders),^8^ some of which can be exacerbated by cannabis use.^18^,^19^ Cannabis can also impact the efficacy of medications, for example, blood thinners, sedatives and antidepressants.^20^ Finally, age-related changes in liver and kidney functions^21^,^22^ can affect the metabolism of cannabis and other medications, increasing the risk of drug interactions and adverse effects.^23^ A recent scoping review found that cannabis use among older adults was associated with greater frequency of mental health issues, problematic substance use and acute health care use, with the harms outweighing any potential benefits and no clear benefit-to-risk ratio.^8^

In this present study, we examined older Canadians’ perceptions of cannabis use and describe the differences between frequent and infrequent consumers, gender-specific preferences and consumers’ concerns. We examined the methods, reasons and motivations for using cannabis, how older adults access cannabis, their perceptions of its harms and benefits and how legalization affected their use patterns and behaviours.

## Methods

***Ethics approval***

Ethics approval was obtained from the Advarra Institutional Review Board (Pro00064863) (Columbia, MD, US). Advarra is a commercial IRB with operations in the United States and in Canada. Advarra complies with the *Tri-Council Policy Statement: Ethical Conduct for Research Involving Humans* (TCPS 2) to ensure adherence to the highest ethical standards. 

Each study participant provided written consent at the beginning of each focus group session.

***Study design***

We used a phenomenological qualitative research strategy to obtain in-depth descriptions of older adults’ cannabis use.^24^ This approach is effective for exploring complex topics as it respects and emphasizes individuals’ lived experiences. We adhered to the Standards for Reporting Qualitative Research.^25^

***Participants***

Individuals aged 60 years and older residing in Canada were recruited through CRC Research, a Canadian firm specializing in qualitative research recruitment. CRC Research used their database, referrals and social media outreach and contacting potential participants via phone. They also posted advertisements targeting specific age groups and locations on Facebook and Instagram (Meta Platforms, Menlo Park, CA, US).

Potential participants were contacted by phone and underwent a screening process to ensure that they met the following eligibility criteria: aged 60 years or older; feeling comfortable speaking in a group setting; and having access to a stable Internet connection and a suitable device. Information was also gathered on participants’ geographical location (province or territory); community setting (rural, urban); gender (male, female, nonbinary or other gender identity); and cannabis use patterns (ranging from never used to infrequent or frequent use, for both medical and nonmedical purposes). 

CRC Research supervisory personnel validated participants by compiling master lists to meet target quotas.

Cannabis use patterns were determined by asking the participants, “Have you ever tried cannabis, either for medical or nonmedical purposes? Would you say… yes, just once, yes, more than once or no?” The participants were then asked, “During the past 12 months, how often did you use cannabis, either for medical or nonmedical purposes? Would you say… never, once or twice, monthly, weekly, daily or almost daily?” We refer to the participants who reported consuming cannabis weekly, daily or almost daily in the past 12 months as frequent consumers; to those who reported consuming cannabis monthly or once or twice in this period as infrequent consumers; and to those who reported not consuming cannabis at all in this period or who had used in the past but no longer do as non-consumers.

Reasons for use were determined based on responses to the following question, “Which of the following best describes the main reason you use cannabis? Would you say… nonmedical (recreational use), medical use with a medical document, medical use without a medical document, or both?” 

***Procedure and data collection***

Data collection occurred over 10 online 90-minute-long focus groups conducted from 22 to 28 September 2022. The focus groups were facilitated by Quorus Consulting Group (Ottawa, ON) under the supervision of a research team member [RG]. A total of 72 participants took part. Each received a CAD100 gift card as compensation for their time and effort. (Detailed participant characteristics are available from the authors on request).

Focus groups were conducted in five regions: Western Canada (British Columbia); Central and Northern Canada (Manitoba, Alberta and the Northwest Territories), Ontario; Quebec; and Atlantic Canada (New Brunswick, Prince Edward Island, Nova Scotia and Newfoundland and Labrador). Each region hosted two focus groups: one for frequent cannabis consumers and the other for infrequent consumers and non-consumers. 

Focus group sessions with participants residing in the province of Quebec were held in French while those in the rest of Canada were held in English. Each focus group included six to eight participants from rural and urban settings and of different ages and genders. Sessions were held using Zoom Workplace (Zoom Communications, San Jose, CA, US), which was also used for observation, recording and transcription. 

Data collection involved open-ended interview questions guided by a moderation guide (available from the authors on request). 

***Data analysis***

To explore participants’ perception of cannabis consumption, we used inductive and deductive thematic analysis methods, as outlined by Braun and Clarke.^26^ The interviews focused on participants’ knowledge, attitudes and perceptions about the benefits and harms of cannabis consumption. These were analyzed using MAXQDA (VERBI GmbH, Berlin, DE).

Three health care researchers [BP, SN, JR] with expertise in substance use and qualitative methods conducted the analysis. Thematic analysis involved systematically identifying themes within the narrative data. The researchers read all the transcripts, discussed initial impressions, extracted relevant phrases and assigned codes to these phrases. These codes were then organized into themes and the themes were reviewed and refined for coherence and distinction.

A final report detailing each theme was prepared, ensuring trustworthiness through peer debriefing. Two team members [BP and SN] conducted the initial analysis and a third [JR] validated the conclusions. An audit trail documented methodological and analytic decisions, with data display tables and visual representations retained.

## Results

Focus group analysis generated 13 subthemes, which were collapsed into five overarching themes: common practices when using cannabis; general knowledge about cannabis; perceived risks and harms of cannabis use; reasons for consumption and the perceived benefits; and stigma and social acceptability post-legalization (Table 1). We examine each of these themes and include quotations from the transcriptions to illustrate the themes.

**Table 1:** Themes and subthemes identified from focus group analysis

Theme / Subtheme	Details / What We Heard
Common practices when using cannabis	
Method of consumption	Three types: oral, inhalation, topical
	Frequent consumers prefer inhalation (familiarity, immediacy of effects)
	Gender differences: males prefer smoking and vaping; females prefer edibles and topicals
Cannabis access	Older adults obtain cannabis products from someone they know, via medical cannabis authorizations, legal cannabis stores on First Nations reserves, online sources, and retail stores
	Frequent consumers obtain cannabis products from online or retail stores; infrequent consumers obtain products from someone they know
	Impact of legalization on cannabis access: legalization appeared to decrease access through friends and improve perceived access to edibles and topicals (among females)
Reasons for cannabis consumption and perceived benefits	
Physical benefits	Managing chronic pain, arthritic pain, aiding sleep, managing withdrawal symptoms, “natural alternative” or “lesser evil” compared to pharmaceuticals
Mental health benefits	Managing anxiety and stress, helping relaxation, and improving concentration
Recreational use	Socialization, overcoming boredom, desire for “high”
	Gender differences: males are more likely to report recreational use
Perceived risks and harms of cannabis use	
Fear of harms to physical health	Fear of unknown adverse effects, concerns about smoking on lung health, fear of cognitive decline and dependency
Fear of penalization	Fear of penalization when travelling with cannabis products
	Concerns about impaired driving, including variability in THC responses, tolerance, product potency, effects duration, and detection accuracy
Risk of mixing cannabis with other substances	Concerns about interactions with alcohol and prescription/nonprescription drugs; personal experiences highlighting risks
General knowledge about cannabis	
Source of information about cannabis	Active sources: health care providers, family, friends, cannabis dispensers
	Passive sources: Internet, TV, social media, scientific sources, personal use
	Desire for unbiased information; low satisfaction with health care providers’ information
	No gender differences observed
Cannabis dosing and product labels	Uncertainty about effective dosages
	Learning via trial and error
	Varied understanding of CBD and THC levels
	Dissatisfaction with labels, need for clearer, more informative labels and supplementary materials
	No gender differences observed
Interactions with health care providers	Perceived lack of knowledge and training among health care providers
	Need for more informed and supportive health care providers
	Mistrust of health care providers’ guidance
Stigma and social acceptability post-legalization	
Stigma related to cannabis use	Persistent stigma despite legalization (frequent consumers)
	No gender differences observed
Social acceptability	Increased acceptability in social circles, more open discussions

**Abbreviations: **CBD, cannabidiol; THC, delta-9-tetrahydrocannabinol. 

***Common practices when using cannabis***

**Methods of consumption**

The focus group participants identified two primary ways for consuming cannabis: oral consumption, which involves ingesting edibles such as gummies, capsules and baked goods (e.g. brownies), and inhalation, which includes vaping and smoking using vape pens, blunts and bongs.

Those who said they preferred smoking cannabis over other forms of consumption often gave “familiarity” and “habit” as reasons. Smoking was more popular among males than females. A few frequent consumers preferred vaping for the immediacy of the “high” (compared to edibles), while some infrequent consumers discontinued vaping because of concerns about unknown adverse health effects.

Some frequent consumers said that they had transitioned from inhalation to oral consumption as they aged to avoid the negative effects of smoking and vaping on their lungs. In contrast, infrequent consumers said that they avoided edibles because of their strength, which could lead to unanticipated “highs.” Female frequent consumers favoured edibles and topicals over smoking to avoid the taste and smell of cannabis and the negative health effects. Males, overall, had no product preferences.

I also stopped smoking … I changed the way that I use cannabis, and it is more appropriate for me too in terms of my lungs. [Frequent consumer] 

Both infrequent and frequent consumers also mentioned using topical applications to manage chronic or arthritic pain. Lotions and oils containing cannabidiol (CBD) and THC were often described as providing relief from pain.

**Access to cannabis**

Older adults obtained cannabis from various sources, including from someone they knew or via medical cannabis authorizations, online stores, legal cannabis stores on First Nations reserves or other legal cannabis retail stores. Frequent consumers obtained cannabis primarily through online stores or dispensaries, while infrequent consumers often mentioned getting cannabis through someone they knew. The infrequent consumers who used cannabis oil for chronic pain typically purchased it from legal cannabis retail stores to ensure its safety.

Frequent consumers noted that legalization had made it harder to obtain cannabis through non-legal sources such as via friends or family because of the increased control and higher prices of legal products:

The black market was going full tilt when it was not legal, and the prices were a lot lower. Again, the minute the government sticks their hands in anything, it’s shot. [Frequent consumer] 

Some female participants, mostly frequent consumers, found that legalization made it easier to obtain edibles and topicals. Male frequent consumers did not mention significant changes in how they obtained cannabis post-legalization.

***Reasons for cannabis use and perceived benefits of use***

**Physical benefits of using cannabis**

Many frequent consumers cited pain management, specifically chronic generalized pain or arthritic pain, as their primary reason for using cannabis. This was also the main reason infrequent consumers gave for using cannabis, particularly CBD in edible or topical form. Many frequent consumers also found cannabis to be an effective sleep aid. Some other participants mentioned that cannabis helped them manage their withdrawal symptoms when tapering off opioids or alcohol. Some infrequent and frequent consumers considered cannabis preferable to pharmaceuticals based on their perception of it having natural properties. Specifically, they described it as a “natural alternative” and the “lesser evil” compared to medications that contain “chemicals.” 

**Mental health benefits of using cannabis**

Many frequent cannabis consumers reported using cannabis to alleviate mental distress, specifically the management of anxiety symptoms and stress levels. A frequent consumer described, “I deal with anxieties, and I can tone myself down with a minute amount of edible.” 

Cannabis helped some participants manage other dependencies. For instance, a frequent consumer stated, “I have an alcohol addiction, so the cannabis helps me to stay away from that.”

Some frequent consumers described positive cognitive benefits, such as a greater ability to relax and improved concentration. Infrequent consumers did not mention the impact of cannabis on any anxiety symptoms, although some did acknowledge its beneficial effects on relaxation and sleep.

**Recreational cannabis use**

Some frequent consumers, mostly males, reported using cannabis for recreational purposes, including to facilitate socialization with other cannabis consumers. They also mentioned that cannabis has helped them overcome feelings of boredom, helping them “pass the time.” Some others used it for pleasure or because they “wanted a high.”

***Perceived risks and harms of cannabis use***

**Fear of harms to health**

Many frequent and infrequent consumers mentioned that a significant reason for avoiding using cannabis was their concern about its effects on their physical health. This fear was more pronounced among infrequent consumers; they were particularly deterred by the unknown adverse effects of cannabis products. Many frequent consumers were also concerned about the impact of smoking on their lungs; some mentioned that they had switched from smoking to vaping or oral consumption to mitigate these risks.

Participants in both groups also expressed concerns about potential cognitive decline due to cannabis consumption. They often linked this fear to their advancing age. One infrequent consumer explained: 

Years ago, I would find, like, if I smoked, and then I went to work the next day, I’d find it hard to be really focused at work on complex problems. And so I’d usually just limit [my use] to the weekends. Now, I don’t know whether it’s because I’m getting older … you begin losing words when you’re talking, you get people’s names wrong, and stuff. [Infrequent consumer] 

The perceived risk of developing a dependency on or getting addicted to cannabis was another concern participants in both groups shared. For example, frequent consumers often mentioned that they no longer “feel a buzz” because of their increased tolerance, which has discouraged them from continuing to use.

Many infrequent consumers described experiences where cannabis had either harmful effects or was ineffective:

I can’t smoke or anything.... Marijuana, when I was young, would make me paranoid. Everything was negative for me. Young people around me, the only thing that they have gotten from it are problems. No car, [poor] mental health. Personally, I have a pretty negative opinion. [Infrequent consumer] 

***Fear of penalization***

Concerns about driving while impaired were prominent among participants in both groups. Most agreed that impairment is unsafe and could lead to severe legal consequences:

Impaired operation of a motor vehicle terrifies me. I don’t care what you’re impaired by, whether you’re sniffing glue, drinking alcohol, or smoking marijuana or eating edibles, impaired driving is a no-no. [Infrequent consumer] 

Many participants remained uncertain about the guidelines regarding cannabis consumption and safe driving. Determining the appropriate waiting period before driving was challenging because of the different consumption methods and individual metabolic rates: 

… It’s hard to tell if someone is high. If someone is drinking and staggering around, you know that. Some people are secret smokers, and if you’re smoking, you’re impaired and you shouldn’t be driving, but people are not good at policing themselves. So, that to me was one of the only negative things about them legalizing cannabis—worrying about the effects when someone was driving. [Frequent consumer] 

Many participants in both groups attempted to conceptualize the effects of cannabis on driving by comparing it to alcohol, and often referenced alcohol-related impaired driving limits to frame their understanding of appropriate cannabis consumption before driving. Discussions often included considerations of individual tolerance: 

It depends on the person and the strength of the joint. Someone said how strong it’s gotten now […] Some people are drunk with one glass of wine and others can drink four, five glasses and they are still standing. [Frequent consumer] 

Both infrequent and frequent consumers expressed concerns about the risk of penalization, particularly when travelling with cannabis products containing CBD or THC. This fear was due to the recency of legalization and their uncertainties about what is legally permitted. Some female frequent consumers reported pausing cannabis use when they started a family, citing the responsibility of motherhood and the potential for penalization. As one female participant explained:

I used it a little bit when I was a teenage hippie, but then I had kids and had to live a responsible life. So, I actually didn’t get around to that, not so much because I thought it would interfere with my mothering, but more because it was a risk if you had kids because back when I was having kids, you could lose your kids if you were caught with pot. [Frequent consumer] 

**Mixing cannabis with other substances**

Both infrequent and frequent consumers expressed concerns about mixing cannabis with other psychoactive substances, such as alcohol, citing a lack of personal knowledge or understanding of potential interactions:

I would be concerned about the effects of alcohol and cannabis on your mental state or if you get impaired because of the additional effect of cannabis and alcohol. [Infrequent consumer] 

Many frequent consumers noted that the choice to mix cannabis and alcohol is subjective and individual tolerance varies:

I think it’s really an individual thing like, you know, to mix or not to mix … Some people shouldn’t mix, period, you know. But some people can do [it] all and it’s fine. [Frequent consumer] 

Some frequent consumers also reported shifting from using alcohol to cannabis as they aged:

I don’t drink at all now … like I don’t have a glass of wine. I have a little bit of a gummy or something like that. [Frequent consumer] 

Participants in both groups also acknowledged the potential harms of mixing prescription and nonprescription drugs with cannabis:

I think mixing any kind of drugs, whether it’s cannabis and other drugs or just other drugs, can be dangerous if you don’t know what you’re doing. [Infrequent consumer] 

Infrequent consumers noted that they were afraid that cannabis could be a gateway drug.

***General knowledge about cannabis***

**Source of information about cannabis**

The participants received information about cannabis from both active and passive sources. Active sources included health care providers, family and friends, and cannabis retailers, while passive sources included multimedia (Internet, TV, social media), scientific articles and personal experience of cannabis use. Both frequent and infrequent consumers most often learned about cannabis during informal conversations with family and friends or from TV and social media. Frequent consumers also mentioned learning about cannabis from cannabis retailers and from personal experience. Very few participants, regardless of frequency of cannabis use, mentioned learning about cannabis from scientific articles. However, some frequent consumers expressed a desire for credible and unbiased information, explaining that it was difficult to find:

I don’t think it’s easy to find unbiased information. They’re either trying to sell it to you or they’re trying to keep you off of it. There isn’t any place that you can get a balanced view. [Frequent consumer] 

For infrequent consumers, having access to unbiased information was not a concern.

**Cannabis dosing and product labels**

Frequent consumers in particular reported uncertainty about the cannabis dosages needed to achieve the desired effects. They often had to resort to trial and error to find the appropriate dosage levels for managing pain effectively. Only a few were confident in their understanding of dosages and the specific effects of different amounts of cannabis.

Frequent consumers varied the levels of CBD or THC depending on their method of use. Those using topicals, such as creams or oils, generally had a better understanding of the CBD levels in products; those using oral or inhalation methods were more familiar with both CBD and THC levels. However, many frequent consumers were unaware of the exact THC content in products and used vague descriptors such as “low,” “medium” or “high.” Among those who mentioned specific numbers in milligrams or percentages, their estimates varied significantly, likely reflecting differences in product types, recall bias and individual interpretation. Frequent consumers were also dissatisfied with the labels on cannabis products, and describing them as uninformative and difficult to understand. Some suggested that supplementary materials, such as information pamphlets, could help clarify the labels and dosages. They also mentioned the need for larger fonts and more detailed dosage information, particularly for edibles:

I don’t understand the labels.... All I know is it’s half CBD, half THC and that’s all.… [If it] says 1000 milligrams ... what does that mean? Maybe there should be pamphlets or something explaining what the labels mean. Instead of just, you know, what’s in the package and the strength of each one. [Frequent consumer] 

How to calculate the dosage. We could start with which ones. An example, gummies, like we spoke of earlier. Is it two? Is it according to your weight? It’s according to what? [Frequent consumer] 

In contrast, infrequent consumers engaged less in the discussions about cannabis product labels and dosages. They said that when they looked at labels, it was primarily to check the cannabis content (THC, CBD or both). They often looked for products containing only CBD and were not unduly concerned about interpreting labels for dosage information.

**Interactions with health care providers**

Few participants (mostly frequent consumers) were satisfied with the information they had received from health care providers. Participants said that they often wished health care providers knew more about cannabis, its health effects and appropriate dosages. The consensus in both groups was that health care providers lacked adequate training on cannabis consumption, which made the participants hesitant about seeking their advice:

I could bring it up with the doctor, but all he’s going to come back with [is] “Well, I’ve done this study, I’ve read this research, I believe in this and we’ve got documented cases.” It’s just going to be hearsay. So, unless you go to someone that has that knowledge [and] that has patients that live it every day, you don’t get an honest answer or the answer won’t be truthful. [Infrequent consumer] 

Many participants thought that health care providers should be better informed about cannabis. Participants in both groups felt that they knew more than their health care providers:

My doctors tend to be similar in age to me, or maybe at most 10 years younger, and they grew up … when it was illegal. So, my knowledge is probably better than theirs. [Infrequent consumer] 

Some participants in both groups mentioned that health care providers were curious about their cannabis use but lacked the necessary knowledge to provide proper guidance:

We didn’t discuss [their] knowledgeability … [They] were just more or less curious as to what my experience has been, so I would say, yes, they probably could stand a little bit of education on that. [Frequent consumer] 

Some frequent consumers described experiencing financial difficulties when trying to obtain prescriptions* for medical cannabis and, consequently, turned to online sources or stores on First Nations reserves, where product purity is uncertain. They often considered going to a walk-in clinic or making an appointment with a doctor, but the reluctance of health care providers to prescribe cannabis for medical use left many seeking alternative sources.

Other participants, in both groups, emphasized the importance of health care providers being open-minded and willing to authorize cannabis, as this would make these consumers less hesitant about discussing their cannabis use with their providers:

I consider it kind of like a supplement or like Tylenol.… Would I tell my doctor I use Tylenol? Unless she asked me, I probably wouldn’t mention it. [Frequent consumer] 

***Stigma and social acceptability post-legalization***

**Stigma related to cannabis use**

Frequent cannabis consumers indicated that although legalization may have reduced cannabis-related stigma among younger individuals, this stigma persists for older adults, and they still feel that they are doing something illegal. One frequent consumer emphasized the need to educate older adults to reduce stigma:

When I was at the senior centre, a lot of [the people there] were against it because we grew up that way, right? Like weed is no good.… Maybe the older population needs to be taught that it’s like a medicine, it’s medicinal, it’s helpful … rather than it’s taboo. [Frequent consumer] 

It is legal today, [but] I still feel weird … I am uncomfortable … [it seems] set in stone that it is illegal. [Frequent consumer] 

Despite the lingering stigma, some frequent consumers felt that there had been a slight shift in attitudes toward cannabis use. They said that they hoped that societal views would continue to improve. In contrast, most infrequent consumers felt that stigma decreased considerably following legalization.

**Social acceptability**

Both frequent and infrequent consumers observed an increase in the acceptability of cannabis use as discussions about it have become more common in their social circles, particularly among friends and family. Some infrequent consumers compared their past experiences with the current openness in discussing cannabis use:

More people are talking about it and suggesting it to their friends. I find that when I’m in conversation with people my age, they say, “Well, I’ve tried this.” [Infrequent consumer] 

## Discussion

Our findings indicate that older adults’ perceptions, knowledge and practices related to cannabis consumption vary based on frequency of use, gender and personal experiences.

Frequent cannabis consumers, who use cannabis weekly or more frequently, do so primarily to manage chronic health conditions like arthritis, chronic pain and insomnia. They also consume cannabis for mental health issues such as stress and anxiety and for improved concentration and relaxation. These findings are consistent with previous research that reported that medical uses or health purposes were the most common reasons for older people using cannabis.^10^,^11^,^27^ A subset of frequent consumers in our study also used cannabis socially and recreationally, for example, to alleviate boredom and enhance social interactions. This “dual purpose” use, which demonstrates the distinct motivations for cannabis use, has previously been observed.^11^,^28^ Concerns arise when individuals who require medical assistance or health care supervision use cannabis recreationally, as this is not tailored to their medical needs. This underscores the complex situations health care providers need to consider when providing medical guidance to individuals who use cannabis for medical and nonmedical purposes.

Data from the 2024 Canadian Cannabis Survey show that the use of dried cannabis by Canadians aged 16 years and older has steadily decreased since legalization, while the use of edible cannabis, including beverages, has increased.^29^ Between 2018 and 2024, the perceived risk of smoking or vaping cannabis increased, while the perceived risk of eating or drinking cannabis did not change.^29^ Despite this reported shift away from smoking and vaping and toward edibles, many of the participants in our study reported that they continue to prefer inhalation methods because these were familiar and because the effects were immediate. Others, however, had made the shift in order to reduce lung-related health risks.

Frequent consumers often learned about optimal cannabis dosages through trial and error because of a lack of clear guidance, which led to inconsistent experiences and potential health risks. They expressed a strong desire for more evidence-based information on dosages and effects. This highlights the need for comprehensive education tailored to the unique needs of older adult frequent consumers to allow them to make informed decisions. Importantly, in line with previous research,^11^ the frequent consumers participating in our study often acknowledged that, while legalization has reduced perceived stigma, especially among younger individuals, it persists among older people. This may be associated with the historical portrayal and legal status of cannabis, which many may have internalized. This stigma might also reflect a generational divide regarding the normalization of cannabis, with youth considering recreational cannabis use an acceptable part of growing up (e.g. experimentational use), while older adults have different life expectations and perceived roles.^30^ Nevertheless, the frequent consumers in our study were optimistic that societal attitudes will continue to evolve favourably. 

Gender differences were evident among frequent cannabis users. Similar to gender differences documented in national surveys and other studies,^10^,^31^-^34^ males were more likely to favour smoking and vaping, while females generally preferred edibles and topicals to avoid the taste and smell of cannabis smoke and the health risks associated with smoking. According to the 2023 National Cannabis Survey, males aged 25 years and older were more likely to use dried cannabis (70.2% vs. 48.4%) than their female counterparts, who more frequently reported using edibles (62.7% vs. 51.9%).^31^ Similarly, although data from the International Cannabis Policy Study show that the dried flower is the product most commonly used by both males and females aged 55 to 65 years, females reported greater use of edibles, oral oils and topicals and males more commonly used the dried flower, hash and solid concentrates.^10^ Females were also generally more cautious than males about the health risks associated with smoking cannabis, and opted for other consumption methods.^32^-^34^

We found that infrequent consumers generally approached cannabis use with greater caution than frequent consumers. They were often discouraged by the lack of information about potential adverse effects and by a fear of dependence on a possible gateway drug. Many infrequent consumers had found cannabis to be either harmful or ineffective at managing their physical conditions. The primary reason for trying cannabis was pain management, and these consumers generally purchased the oil from a retail store to be sure it was safe. Infrequent consumers did not emphasize the mental health benefits of cannabis as much as frequent consumers did, although some acknowledged that cannabis helped them relax and sleep. 

In contrast to frequent consumers, infrequent consumers generally perceived that the stigma associated with cannabis use had decreased following legalization. This group may consider the legal changes “liberating,” as these allow them to explore cannabis use without the moral and legal repercussions that may have inhibited them in the past. This difference in perception could be attributed to varying degrees of exposure. Frequent consumers, with a longer history and perhaps a deeper understanding of the implications of cannabis use, may have still been dealing with deep-rooted stereotypes and personal reservations. Infrequent consumers, potentially newer to the cannabis experience and therefore less troubled by past prohibitions, might merely perceive the benefits of the recent changes in the law.

Despite differences in usage patterns and motivations, frequent and infrequent consumers shared some common perceptions. Some viewed cannabis as a natural alternative to pharmaceuticals, and therefore a “lesser evil” compared to conventional medications, a perspective also observed in other studies.^27^,^28^ However, there is insufficient high-quality clinical evidence showing that cannabis is effective in the treatment of most health conditions and particularly as a first-line treatment.^35^ A systematic review and meta-analysis of randomized clinical trials concluded that medical cannabis and cannabinoids—both prescription cannabinoids and plant-based preparations—provide few benefits in the management of chronic non-cancer and cancer-related pain, with the quality of evidence low.^36^ Other research has shown that the effectiveness of cannabinoid products such as THC and CBD and pharmaceutical formulations with standardized THC to CBD ratios (e.g. nabiximols, dronabinol, nabilone) have limited and inconsistent effectiveness in treating mental health disorders such as depression and anxiety.^37^-^39^

Consequently, the College of Family Physicians of Canada recommends limiting the use of medical cannabinoids in general and restricting their use for neuropathic pain, palliative care, chemotherapy-induced nausea and spasticity due to multiple sclerosis or spinal cord injury.^40^ For example, nabilone (Cesamet)^41^ is approved for severe nausea and vomiting as a result of cancer chemotherapy, nabiximols (Sativex)^42^ for spasticity in multiple sclerosis and CBD (Epidiolex)^43^ for certain treatment-resistant childhood seizure disorders. Research examining cannabinoid efficacy for conditions like pain, anxiety, mood disorders, psychosis, neurodegenerative disorders and substance use disorders is ongoing.^44^

Concerns remain about the risks of cannabis dosing, adverse effects and interactions with existing medications in older populations. Both infrequent and frequent consumers were worried about mixing cannabis with prescription and over-the-counter drugs, explaining that they were often unaware of potential interactions. Because older adults may be managing comorbidities with various medications, there is a need for greater awareness of and more education about possible interactions.^45^ In addition, many older adults may assume the potency of cannabis products to be similar to that of the cannabis they used decades ago. The higher THC levels of currently available products pose specific risks, especially for people with existing health conditions or receiving multiple medications, potentially causing complications such as increased heart rate, elevated blood pressure, anxiety and disorientation.^14^-^17^ Another and often overlooked concern is that many CBD products contain trace amounts of THC, and consuming high doses of CBD may lead to sufficient THC exposure to result in intoxication or impairment.

Both frequent and infrequent consumers described having social and societal fears, such as the fear of penalization, particularly regarding travelling with cannabis products and impaired driving laws. Consumers in both groups were confused about how impairment is assessed. While THC concentration limits for impaired driving exist in Canada, the participants felt that these limits do not account for individual differences in tolerance, product potency, method of consumption and duration of effects. These concerns demonstrate the need for improved tools to accurately assess impairment, as well as targeted public education to raise awareness about cannabis use and impaired driving.

Another concern was confusion over cannabis product labels, with many study participants describing labels as difficult to understand. This finding aligns with data from the International Cannabis Policy Study,^10^ which reported that most consumers’ comprehension of THC levels in cannabis products is low. This emphasizes the need for clearer, more informative labelling.^46^-^48^ This is particularly important given the legal context and the variety of new products available on the market. The Expert Panel on the Legislative Review of the *Cannabis Act* recently recommended improvements such as simplifying THC and CBD displays, allowing transparent packaging for dried flowers and using QR codes for detailed product information.^49^ Implementing a standard THC unit in product labelling and packaging, and as part of consumer education, has also been suggested.^50^ Ensuring the accuracy of product labelling is also crucial for promoting informed and safe consumption. Inconsistencies in the labelling of legal cannabis oil products sold in Ontario indicate a need for greater quality control, as variations may affect the ability of consumers to make informed choices.^51^

Consistent with other research,^11^,^52^-^55^ the older adults participating in our study often noted gaps in health care providers’ knowledge about cannabis. Some participants felt that they knew more than their health care providers about cannabis, which affected their confidence in the guidance they received. The participants emphasized the value of having informed and supportive health care providers to help them safely navigate cannabis use. Enhanced education and training for health care providers, including nurse practitioners, was seen as important for addressing questions about the therapeutic benefits, appropriate dosages, potential interactions with other medications and possible adverse effects of cannabis. 

While a significant number of older adults use cannabis for medical purposes, or are interested in doing so, the lack of evidence supporting the efficacy of cannabis as a treatment for many of the conditions it is commonly used to manage (with the exception of neuropathic pain^16^,^40^,^56^) makes it difficult to provide validated guidance, particularly as older consumers often use cannabis for both medical and nonmedical purposes. This highlights the need for more research into medical cannabis. At the same time, cannabis and cannabinoids should not be considered the only solution to the mental and physical health issues older adults frequently experience. Greater attention is needed to address the high prevalence of these health challenges, along with increased investment in a range of effective treatment options, irrespective of whether these include cannabis and cannabinoids.

***Limitations and strengths***

Our study had some limitations. First, as an exploratory investigation, it provides a focused snapshot of frequent and infrequent cannabis consumers among older adults, which limits the generalizability of the findings to the broader population of older adults in Canada who use cannabis. Participants were recruited from a consumer panel database built through ad campaigns, referral programs, targeted recruitment initiatives aimed at reaching hard-to-reach populations and other methods. Although this recruitment strategy enhances diversity, the participants may not represent the range of people in the older population in Canada and our findings may not capture the challenges, perspectives and behaviours of individuals from rural areas, with lower socioeconomic status or different cultural contexts. In addition, it is possible that individuals who choose to join such panels have unique characteristics, beliefs or behaviours that could influence findings. 

Second, the use of focus group methodology may have introduced self-selection bias, as participation was voluntary, potentially narrowing the diversity of views and experiences represented. 

Third, participants may also have moderated their views in a group setting, either choosing not to divulge certain information or aligning their opinions with others to avoid disagreement. 

Finally, we examined gender within a binary framework (males and females) as none of the participants identified as nonbinary or as having another gender identity. Consequently, the findings may not fully capture the perspectives and experiences of older adults with diverse gender identities. Similarly, the majority of participants identified as White, limiting the generalizability of the findings to other ethnicities. Future research should aim to explore perceptions of cannabis use across a broader spectrum of gender identities and among individuals from different ethnic backgrounds. 

Nevertheless, our study has several notable strengths. It is one of the first to specifically address cannabis use by older adults, a population that is underrepresented in this research field. Our research addresses critical gaps in the literature by exploring the unique experiences, motivations, behaviours and perceptions of cannabis consumption in this population post-legalization. By differentiating between frequent and infrequent cannabis consumers, our research adds to the current knowledge by identifying how older adults integrate cannabis into their health care and social lives. Our findings also highlight important gender differences in consumption preferences and perceptions of health risks, contributing to a more comprehensive understanding of cannabis use patterns. 

Finally, the qualitative nature of this research allowed for in-depth exploration of individual experiences, offering rich data that can inform future research, health promotion initiatives and policy decisions aimed at this growing and important population.

## Conclusion

Our study contributes significantly to what we know about cannabis use among older adults in Canada. We emphasize the diverse reasons for use, methods of consumption and varying perceptions of cannabis-related benefits and risks, as well as binary gender differences in these patterns. As the landscape of cannabis legalization continues to evolve, it is essential to prioritize the needs and experiences of older adults in cannabis research and policy. Providing targeted education, clear guidelines and supportive health care environments can help mitigate the risks of cannabis consumption in this population.

## Acknowledgements

We acknowledge Quorus Consulting Group for their professional support in facilitating the focus groups.

## Funding

This research received funding from the Canadian Centre on Substance Use and Addiction (CCSA), which was made possible through the Cannabis Research Initiative, Substance Use and Addictions Program, Health Canada.

## Conflicts of interest

None to declare.

## Authors’ contributions and statement

JR: Conceptualization, methodology, supervision, visualization, writing—original draft, writing—review and editing.

BP: Formal analysis, writing—original draft. 

SN: Formal analysis, writing—original draft. 

EW: Writing—review and editing.

NC: Writing—review and editing. 

RG: Conceptualization, methodology, visualization, writing—original draft, writing—review and editing. 

All the authors provided feedback, contributed to the revisions and approved the final manuscript. 

The content and views expressed in this article are those of the authors and do not necessarily reflect those of the Government of Canada.
